# Analysis of Nutrient-Specific Response of Maize Hybrids in Relation to Leaf Area Index (LAI) and Remote Sensing

**DOI:** 10.3390/plants11091197

**Published:** 2022-04-28

**Authors:** Atala Szabó, Seyed Mohammad Nasir Mousavi, Csaba Bojtor, Péter Ragán, János Nagy, Attila Vad, Árpád Illés

**Affiliations:** 1 Faculty of Agricultural and Food Sciences and Environmental Management, Institute of Land Use, Engineering and Precision Farming Technology, University of Debrecen, 138 Böszörményi St., H-4032 Debrecen, Hungary; szabo.atala@agr.unideb.hu (A.S.); nasir@agr.unideb.hu (S.M.N.M.); ragan@agr.unideb.hu (P.R.); nagyjanos@agr.unideb.hu (J.N.); illes.arpad@agr.unideb.hu (Á.I.); 2 Institutes for Agricultural Research and Educational Farm (IAREF), Farm and Regional Research Institutes of Debrecen (RID), Experimental Station of Látókép, University of Debrecen, H-4032 Debrecen, Hungary; vadattila@agr.unideb.hu

**Keywords:** factor analysis, LAI, maize, regression analysis

## Abstract

Leaf area index (LAI) indicates the leaf area per ground surface area occupied by a crop. Various methods are used to measure LAI, which is unitless and varies according to species and environmental conditions. This experiment was carried out in three different nitrogen ranges (control, 120 kg N ha^−1^, and 300 kg N ha^−1^) + PK nutrient levels, with five replications used for leaf area measurement on seven different maize hybrids. Hybrids had different moisture, protein, oil, and starch contents. N (1, 2) + PK treatments had a desirable effect on protein, starch, and yield. P0217 LAI had a minimal response at these fertiliser levels. LAI for Sushi peaked at different dates between control and fertiliser treatments. This result showed that Sushi has an excellent capacity for LAI. LAI values on 15 June 2020 showed minimum average values for all hybrids, and it had a maximum average values on 23 July 2020. LAI had maximum performance between the average values treatments in Sushi, Armagnac, Loupiac, and DKC4792 on 15 June 2020. This study also provides insights for examining variably applied N doses using crop sensors and UAV remote-sensing platforms.

## 1. Introduction

Maize is used to feed people and animals. The component percentages of maize are 66.70% starch, 10% protein, 4.8% oil, 8.5% fibre, 3% sugar, and 7% ash [1]. The LAI plant canopy analyser is used to perform plant photometric measurements and estimate plant biomass for precision crop production. Nitrogen affects the leaf area index, and examining its level can help predict crop output. The leaf area index contributes to climate modelling, field-level yield estimation, and crop yield prognosis. Remote sensing support significantly contributes to estimating LAI at local, district, and global scales [2]. One of the most important variables required to estimate crop production and global climate testing is the leaf area index (LAI) [3]. The quantitative measure of foliage density is shown by the leaf area index, which helps in monitoring the different stages of development [4]. Genotype, climate and soil factors affect the leaf area index [5,6]. At a foliage density of 300 kg N ha^−1^, a leaf area index of 5.1 was measured [7]. There was a significant correlation between LAI and yield (r = 0.91 **), as well as regression [7]. Hammad et al. [8] found that the highest value (5.06) was measured at a foliage density of 250 kg N/ha in their experiment. In addition to maize grain yield and its components, the development stages of maize slow down with low N supply. Moreover, reduced N decreases harvest index and leaf area-index values [9]. Biomass can be determined based on leaf area and is influenced by the plant population and nutrient supply [10]. In addition to grain yield, nitrogen stress also reduces kernel number [11]. A high N (225 kg ha^−1^) contributed to reaching an LAI of 4.05 at the silking stage. There is a significant positive correlation between LAI and yield. Application of 225 kg ha^−1^ N increased the thousand-kernel weight, leaf area index, amount of assimilates, and grain filling period [12]. Hokmalipour et al. [13] state that the application of nitrogen had a positive effect on the chlorophyll content, leaf area index, leaf dry weight, and grain yield of maize hybrids. According to Sharanabasappa and Basavanneppa [14], significantly high grain yield (8023 kg ha^−1^) was achieved with 225: 112.5: 56.25 NPK kg ha^−1^ doses, with the best yield component values and higher leaf area index corresponding at this NPK level. Exploring LAI is fundamental both in space and time for agricultural applications, such as when estimating yields and monitoring growth rates [15,16]. Elings [17] examined LAI differences at three nutrient levels. An LAI of 3.6 was obtained at a nitrogen dose of 150 kg ha^−1^, but this did not differ significantly from the LAI at a nitrogen dose of 120 kg ha^−1^. Furthermore, there was no significant difference between the 120 and 90 kg ha^−1^ N doses. However, a significant difference was found between 150 kg ha^−1^ and 90 kg ha^−1^ (*p* < 0.05). Higher leaf area index was measurable due to the higher N dose [18]. The interception of light by the leaves, their transpiration, and the determination of the accumulation of dry matter are important in order to perform an appropriate simulation, as they affect the development and production of the plant [19,20,21]. The LAI model starts with slower growth, followed by faster growth, until the maximum LAI is achieved as the plant leaves senescence and the plant reaches physiological maturity, after which the LAI decreases [22]. LAI increases from the closed plant stock stage to the stem elongation stage to the 12 leaves stage and peaks during the silking period and has an effect on dry matter accumulation [23]. Leaf area is affected by nitrogen supply [24]. One study showed that there were significant differences in grain yield between 0 kg ha^−1^ N and 225 kg N ha^−1^ treatments (*p* < 0.05) [25]. With continuous growth of the plant stock, the leaf area index also increases. There was a significant difference at 90 and 225 kg ha^−1^ N compared to the control treatment. At the end of the vegetation period, the LAI was 4.5 for 90 kg ha^−1^ N and 5.5 for 225 kg ha^−1^ N, with less than 3 LAI for the nitrogen-deficient control [26]. There is a linear relationship between LAI and nitrogen supply [27]. Increased LAI leads increased maize dry matter production [28]. One experiment showed that leaf area index influences the components of the crop during dry matter accumulation [29]. Another experiment showed correlation between percentage decrease in leaf area and decrease in grain yield [30]. A lower leaf area index accompanied nitrogen deficiency, with lower grain yields [11]. By increasing N while keeping PK constant, the examined hybrids were more resistant to environmental stress; additionally, the protein content increased, especially during the wet season [31]. Similarly, based on the results of Horváth et al., [32] found that, in addition to the optimal amount of fertiliser, the application time of the fertiliser determines the yield of hybrids. In drought conditions, nitrogen was most effective at 150 kg ha^−1^ N (V6 phenophase) for Sushi and 120 kg ha^−1^ N (V6 phenophase) for Fornad. There has been a breakthrough in precision agriculture. The use of satellite data is widespread, and is implemented in mapping cropland [33], estimating nitrogen requirements for plants [34], monitoring health status, and predicting crop yields [35]. Using NDVI measurements for corn biomass and grain yield can help farmers make in-season agricultural management decisions. Indirect measures of spectral reflectance and leaf area index were used to estimate forage biomass and grain yield in Virginia [36]. Analysis and evaluation of the dynamic changes and heterogeneity of canopy cover and NDVI were aided by properties extracted from unmanned aerial vehicle high throughput phenotypic platform (UAV-HTPPs) images of plots populated by different genotypes [37]. The vegetation index was correlated with the yield. NDVI is the normalized vegetation index, and the enhanced vegetation index is EVI [38]. NDVI can be calculated using Equation (1) [39]:

(1) $$NDVI=\frac{\rho nir-\rho red\;}{\rho nir+\rho red}$$

UAVs have the ability to measure NDVI, and NDVI dynamics during the growth phase can be analysed [40,41]. One test using a UAV found an NDVI range from 0.14–0.88 [42]. UAV-acquired NDVI data can be used to produce yield maps [43]. UAV imaging data has high correlation with NVDI (r^2^ = 0.89), which makes it suitable for estimating chlorophyll content, which helps monitor crop production [44]. In the R1 phase, a correlation of 0.72 was found between NDVI based on UAV image data and maize grain yields ranging from 1.9–4.6 t/ha [45]. There is a close relationship between UAV data, NDVI, and crop biophysical variables. Statistical analysis found r^2^ = 0.95 between UAV, NDVI, and LAI. This close relationship supports the fact that the remote sensing using UAVs is reliable for estimating LAI and percent canopy cover [46]. A close correlation (r^2^ = 0.601–0.809) between NDVI and fertilizer levels for both rice and wheat has also been identified [38].

## 2. Results

The performed variance analysis showed that hybrids have significant variations in moisture, protein, oil, and starch. NPK fertiliser treatments had a significant effect on protein, starch, and yield. The sampling date had no significant effect on the examined parameters. LAI had a significant effect on moisture in this study. However, LAI did not have a significant effect on protein, oil, starch, or yield (Table 1).

**Figure 1:**
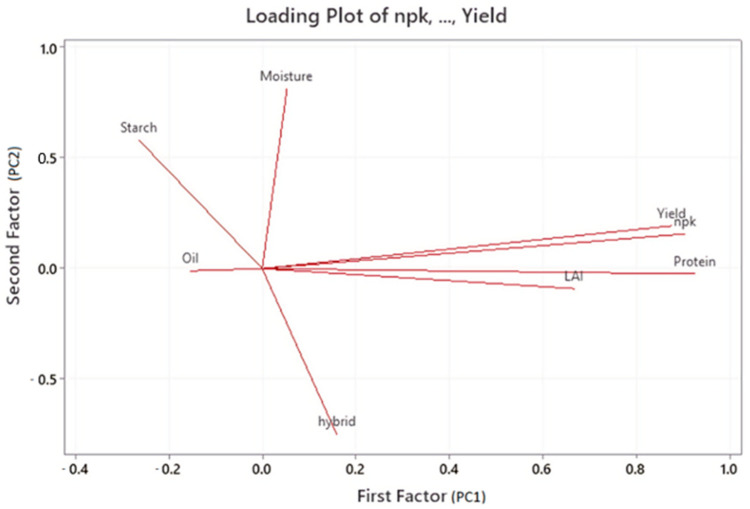
Factor analysis biplot on yield parameters.

The performed factor analysis showed that the first factor includes N(1, 2) + PK levels, protein, LAI, and yield. These parameters covered 37 percent of all data, i.e., these parameters have a joint effect. However, in the biplot, protein and LAI had a negative effect on N(1, 2) + PK and yield. A positive effect was observed between yield and N(1, 2) + PK treatments, protein, and LAI. The second factor includes moisture and starch, covering 27 percent of all data. Moisture and starch had a negative effect on the first factor and positive on the second factor, i.e., they had positive effects based on the biplot. The third factor includes oil and hybrids, covering 17 percent of the total data (Table 2, Figure 1).

Regression analysis showed significant effects on yield, starch, oil, protein, and moisture. Yield and protein were affected by LAI with N(1, 2) + PK. Maximum oil and starch were obtained by reducing N(1, 2) + PK fertiliser levels. Reducing LAI increased grain moisture. Regression analysis showed variations in yield, starch, oil, protein, and moisture between species were minor (Table 3).

**Figure 2:**
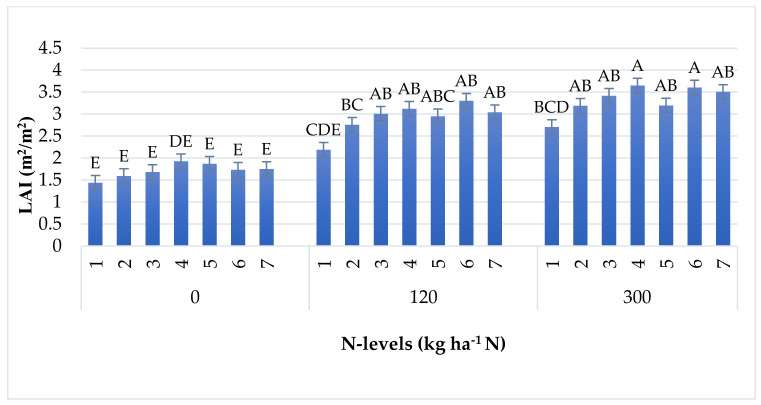
Interaction effect of N(1, 2) + PK on LAI between hybrids. Treatments with the same letter are not significantly different.

Fertiliser was shown to have the maximum effect on LAI in Sushi and Loupiac at 300 kg ha^−1^ N. Lesser gains were found in SY Minerva, Fornad, Armagnac, and DKC4792 at 300 kg ha^−1^ N and Fornad, Sushi, Loupiac, DKC4792 at 120 kg ha^−1^ N. SY Minerva had the same performance at 120 kg ha^−1^ N as P0217 at 300 kg ha^−1^ N. Correspondingly, P0217 at 120 kg ha^−1^ N had low performance in terms of LAI. All hybrids had the same LAI at the control level except for Sushi, which was higher (Figure 2).

**Figure 3:**
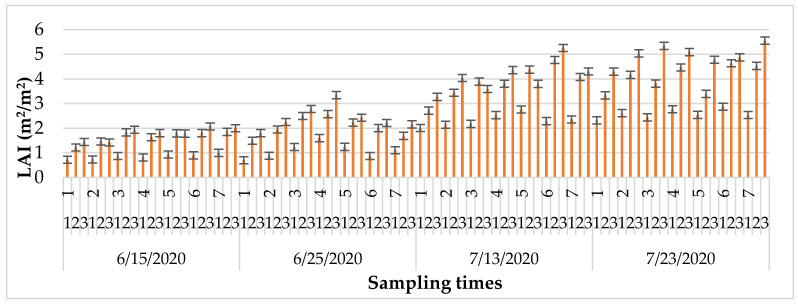
Interaction sampling time in N(1, 2) + PK on LAI between hybrids.

Interaction sampling time in N(1, 2) + PK in hybrids showed that the minimum LAI was on 15 June 2020, and the maximum was on 23 July 2020. Maximum LAI was measured in Fornad at 300 kg ha^−1^ N on 15 June 2020. Sushi showed maximum LAI at the 300 kg ha^−1^ N on 25 June 2020, Loupiac showed maximum LAI performance at the 300 kg ha^−1^ N on 13 July 2020, and Fornad and DKC4792 showed maximum LAI performance at the 300 kg ha^−1^ N on 23 July 2020. LAI showed maximum performance in Sushi, Armagnac, Loupiac, and DKC4792 on 15 June 2020. Fornad and Sushi hybrids had a top performance on 25 June 2020, Sushi and Loupiac had a maximum performance on 13 July 2020, and SY Minerva and Loupiac had a complete performance on 23 July 2020 on 120 kg ha^−1^ Ns. LAI had a complete performance in DKC4792 on 15 June 2020; Sushi had a maximum performance on 25 June 2020; Armagnac had a maximum performance on 13 July 2020; Loupiac had a maximum performance on 23 July 2020 on the control fertiliser treatment (Figure 3).

**Figure 4:**
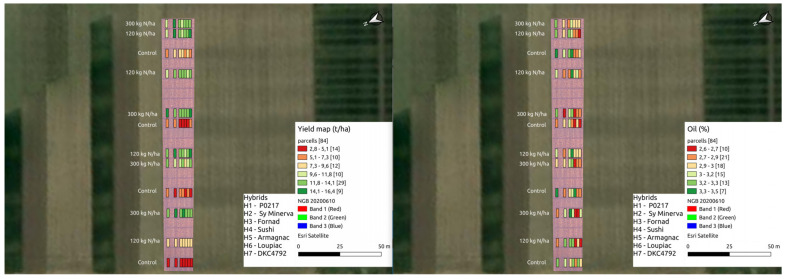
Yieldvalues on the drone map.

**Figure 5:**
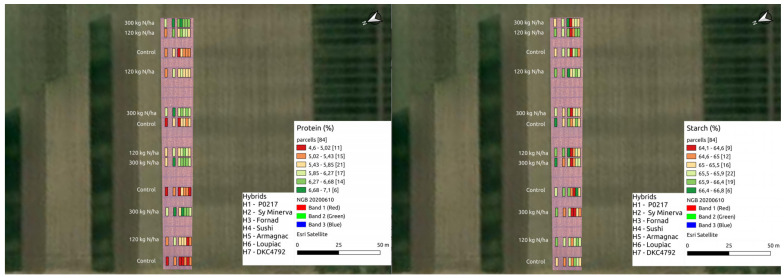
Yield parameter values on the drone map.

**Figure 6:**
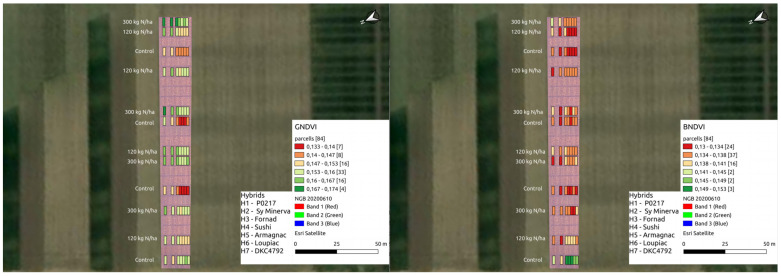
Vegetation index values on the drone map.

BNDVI, ENDVI, GNDVI, BNDVI*ENDVI, GNDVI*ENDVI and BNDVI*GNDVI*ENDVI had no significant effects on oil or starch. BNDVI, ENDVI, GNDVI, BNDVI*ENDVI, GNDVI*ENDVI and BNDVI*GNDVI*ENDVI had significant effect on yield and protein (Table 4, Figure 4, Figure 5 and Figure 6).

The parameters were based on Least Significant Difference test (LSD). Analysis showed that yield was highest for SY Minerva and lowest for P0217 and Armagnac. DKC4792, Sushi, Fornad, and Loupiac had similar yields. Sushi and P0217 also had similar yields. Loupiac, Armagnac, and DKC4792 had similar oil content. Protein was highest in SY Minerva and lowest in P0217. Fornad, Loupiac, and P0217 had similar protein content. Sushi had the lowest starch content. Regarding vegetation indices, BNDVI was highest for Fornad and lowest for SY Minerva. Fornad and Loupiac had similar BNDVIs. DKC4792, Sushi, and Armagnac also had similar BNDVIs. P0217 had the highest GNDVI. DKC4792, Loupiac, Armagnac, and Sushi had similar GNDVIs (Table 5).

Statistical analysis showed differences between nitrogen levels. Yield, protein, and GNDVI were highest at 150 kg ha^−1^ N. Starch was highest at 60 kg ha^−1^ N. ENDVI was highest at 0 kg ha^−1^ N (Table 6).

## 3. Discussion

LAI is important for determining the percentage of solar radiation absorbed by each plant, which affects plant growth and final dry matter yield. The growth and differentiation of vegetative and reproductive organs occurs during the growing season. These processes determine biomass production and distribution, and, most importantly, the creation of economically important crops [47]. Therefore, yield formation should consider all factors and processes associated with total biomass production and its economically important component, usually grain yield. A great deal of biological and agronomic information exists about each of factors maximizing foliage size and/or activity. Understanding the interrelationships between all factors and considering the response of a complex vegetation production system to changes in any of the elements is essential for successful farming [48]. The result showed that hybrid maize varieties had differences in moisture, protein, oil, and starch. N(1, 2) + PK treatments had a desirable effect on protein, starch and yield. Sampling time did not have any effect on the examined parameters, but LAI had various correlations with moisture in this study. Factor analysis showed that N(1, 2) + PK and yield were positively correlated, i.e., increasing N(1, 2) + PK increases yield. LAI had an effect on yield, N(1, 2) + PK, and protein. LAI can influence protein and yield, i.e., N(1, 2) + PK and LAI had the highest effect on maize performance. The result of LAI showed that maize hybrids had a different capacity of LAI at different fertiliser levels. Fertiliser levels can affect LAI, but it depends on the capacity of the maize hybrid. This study showed that 120 kg ha^−1^ N to 300 kg ha^−1^ N resulted in the same LAI for P0217. Therefore, P0217 LAI responds minimally to variations in fertiliser level. Sushi LAI was maximum at different times for control vs. fertiliser treatments. This shows that Sushi has an excellent capacity for LAI. Fertiliser can improve LAI in maize hybrids. Increasing the amount of nitrogen fertiliser causes a high leaf area index. Other researchers have reported similar results on the positive effect of nitrogen fertiliser on maize and wheat [6,49,50,51,52,53,54]. Nitrogen had positive effects on green bean plants, improving the plant’s physical performance by increasing the leaf area index. Since the maximum leaf area index occurs at the time of flowering, the higher the leaf area, the more solar radiation the plant can use and the more photosynthetic material it produces. Finally, LAI affects the seeds in the stem and the grain yield [55]. Leaf area index (LAI) is one of the most important indicators of plant growth and crop yield. Therefore, monitoring this index’s spatial and temporal distribution in agricultural fields can indicate how to apply farm management strategies such as irrigation and uniform water distribution. The amount of nitrogen is one of the practical factors influencing leaf-surface development of each plant, and, consequently, the development of plant shading in maize. Increasing the size and longevity of each vessel increases the leaf area index [56]. Plants develop greater leaf area with more nitrogen. The increase in leaf area index can be attributed to the rise in the green area of the plant, which determines the photosynthetic capacity of the plant [57]. In general, a high leaf area index due to more nitrogen application is due to the positive effect of this element on leaf size and longevity [58]. With increasing soil nitrogen, leaf area index expansion increases, while light penetration into the canopy and light consumption efficiency also increase. Therefore, crop growth rate, leaf area index, and grain yield increase [26]. Nitrogen is one of the essential agronomic factors that significantly affects growth indices. Selecting the appropriate amount of nitrogen fertiliser can achieve a balanced combination of growth indices in the plant and improve crop yield [58]. In some cases, experimental results confirm the positive relationship between LAI and grain yield, but in other cases reject it. LAI is partly due to the presence of green fibres active in photosynthesis in organs other than the leaves, which may not be considered when estimating LAI. The maize spike provides a significant portion of photosynthetic material, and the whole active surface of photosynthesis takes on a different dimension [59,60,61,62,63]. The results showed that there was a difference between hybrids. SY Minerva had the highest yield. Sushi and P0217 had the highest oil content. SY Minerva had the highest protein. For vegetation indices, Fornad had the highest BNDVI and ENDVI, and P0217 had the highest GNDVI. Based on LSD, different nitrogen doses affected yield, yield parameters, and vegetation indices. Karki [64] observed significant effects due to genotype and nutrient level on NDVI at different growth stages. A positive and strong correlation was found between NDVI and grain yield. In their study, for plant N uptake at the V10–V12 and V6–V12 stages, GNDVI and CIgreen demonstrated the importance of red-edge vegetation indices for estimating summer maize N status. This study also provided insights for in-season variable-rate N management using commercial active crop sensors and newly launched satellite remote-sensing platforms with red-edge bands [65].

## 4. Materials and Methods

The experimental plants were seven maize (*Zea mays* L.) hybrids with different FAO numbers and groups of maturity. The experiment was carried out at the Látókép Experiment Site of the University of Debrecen (47°33′ N, 21°26′ E; 111 m asl). The experimental station was located in high-quality calciferous chernozem soil, with width top (80 cm) A layer. The average organic matter in the plots was 2.13% in the top 30 cm. The pH content decreased slightly with increasing nitrogen levels. The average soil pH was slightly acid (5.80) (Table 7).

The experiment was established in 1983 and has been continuing with unchanged parameters for 38 years using the same nutrient replenishment scheme, location, soil tillage, and agrotechnical support. The experiments were carried out in two different nitrogen ranges (120 kg N ha^−1^, 300 kg N ha^−1^), and phosphorus and potassium were applied to each plot in the form of the same autumn basic fertiliser (P_2_O_5_: 184 kg ha^−1^, K_2_O: 216 kg ha^−1^); the total negative control has been used for measurements without artificial and organic nutrient replenishment for 38 years. The measurements were carried out in the 2020 season.

Soil tillage was carried out with winter ploughing on 25 October 20219, and secondary tillage with preparation of the seed bed was carried out on 9 April 2020. The sowing date was 17 April 2020, with 73.000 plant ha^−1^ density and 6 cm sowing depth. Herbicide treatment containing 345 g l^−1^ tembotrione, 68 g l^−1^, thiencarbazone-methyl, and 134 g l^−1^ isoxadifen-ethyl was applied in a dose of 0,3 l ha^−1^ on 18 May 2020. Mechanical weed control was applied on 27 May 2020. The harvest date was 23 October 2020, the harvesting process was done with a plot size harvester (Sampo SR2010, Sampo Rosenlew, Finland). The yield quality parameters (protein, starch, oil, and moisture content) were measured on the plot-level with near-infrared transmittance (NIT) technology (Perten DA 7250, PerkinElmer Ltd., Waltham, MA, USA).

The experiment was a two-factor field experiment with a strip-plot design and four replications, allowing for appropriate statistical evaluation. Five replications were used for leaf area measurement (LAI). Five measurements were taken per row in each plot. The first measurement was next to the left row, then three measurements were distributed proportionally in the row, and the fourth measurement was taken on the right, after which the values were averaged. Leaf area measurements were carried out with the SS1 SunScan Canopy Analysis System (Delta-T Devices Ltd., Cambridge, UK) The SS1 SunScan can measure the difference between two plots in the same sampling time. The same ELADP value was used at the time of measurement and at treatment. Sampling was performed 4 times during the growing season. Sampling times were based on heat sums and phenological phases—1st sampling (15 June 2020): 6 leaves −321.7 °C heat sum; 2nd sampling (25 June 2020): 10 leaves −493.1 °C heat sum; 3rd sampling (13 July 2020): 14 leaves −758.4 °C heat sum; 4th sampling (23 July 2020): silking −896.1 °C heat sum.

Based on the manufacturer’s recommendation for hybrid breeders, the tested hybrids have the following parameters: DKC4792 (7) has a fast water release ability and drought tolerance; P0217 (1) is drought tolerant; SY Minerva (2) is characterised by a strong and stable stem; Fornad (3) has a good nutrient management; the initial vigour of Sushi (4) reached the maximum score on a scale of 9. Armagnac (5) has better-than-average adaptability to sowing date, as it responds to delayed sowing with relatively less yield loss than other hybrids in similar maturity groups. Finally, Loupiac (6) has above-average grain weight, and its thousand-kernel weight reaches 400 g. The examined hybrids account for a remarkable part of Hungarian and European maize farming.

**Figure 7:**
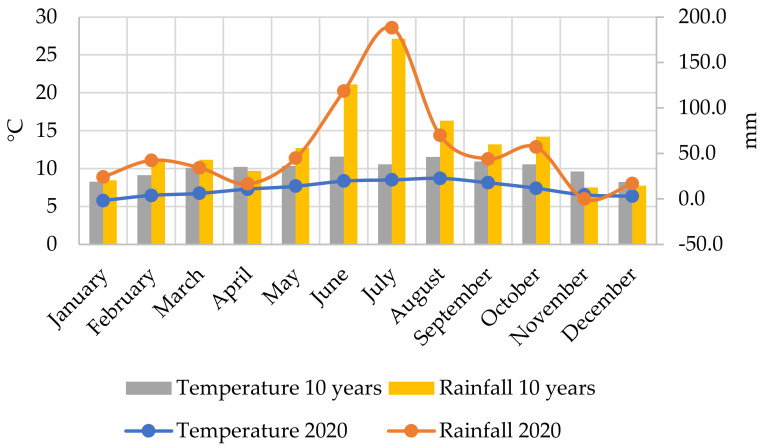
Average rainfall and temperature values in 2020 and the last 10 years, Látókép CropProduction Experiment Site, University of Debrecen.

The year 2020 showed great similarity to 2016, especially in terms of the distribution of precipitation conditions. In both years, a severe rainfall deficit in the spring was followed by outstanding monthly amounts for the three months of summer. As a result of the average or slightly positive precipitation anomaly in the autumn, the amount of precipitation during the entire growing season was significantly higher than in 2016, resulting in a surplus of nearly 180 mm. Regarding air temperature, a significant negative temperature anomaly in May was obvious, which, coupled with the lack of spring precipitation, had a negative effect on plant development during the sowing and germination period. As for the summer months, June and July were average or slightly cooler than average, while August was clearly warmer than average, a trend that continued into autumn. The abundant rainfall around the flowering period and the moderately high temperature conditions at this critical stage of development probably contributed to the development of a good crop (Figure 7).

ANOVA is a statistical test to determine the difference between the means of two or more independent statistical populations. In other words, variance analysis is used to compare two or more groups to see if there are significant differences. Factor analysis is used to decrease many variables into fewer factors. This method extracts the highest common variance from all variables and puts them into a standard score. We can use this score to estimate all variables for further analysis. Linear regression creates a linear model between the “Response” variable and one or more “Explanatory” variables. Regression is often used to discover a linear relationship between variables. In this case, it is assumed that one or more descriptive variables whose value is independent of the other variables or under the researcher’s control can effectively predict the response variable, whose value does not depend on the explanatory variables under the control of the researcher. The purpose of regression analysis is to identify the linear model of this relationship. Factor analysis is one of the multivariate methods in which independent and dependent variables are not considered. This method is considered an interdependent technique, and all variables are interdependent. Factor analysis plays an important role in identifying latent variables or the same factors through observed variables.

The corn experiment was recorded with a DJI Phantom 4 Agro drone. The flight altitude was 40 m with a spatial resolution of 1.75 cm/pixel. The ortho photo was created from the raw images using WebODM. The drone is equipped with a colour-filtered NGB camera so it captures images in NIR, green, and blue channels. The following vegetation indices were created from the 10.06.2020 images:

BNDVI = (N − B)/(N + B)

GNDVI = (N − G)/(N + G)

ENDVI = ((N + G) − (2 × B))/((N + G) + (2 × B)).

A mask was created from the BNDVI record of the dataset for values above 0.09 and used to cut the GNDVI and ENDVI records. The experimental plots were digitized, and then plot-level vegetation index values free of soil disturbance were obtained in shape format using the Quantum GIS Zonal Statistics module.

The attribute table of the shape format was used to create the numerical database for statistical analysis. From this, a strip-plot analysis of variance was carried out, followed by a post hoc least significant difference (LSD) test for comparison of means.

Linear and multilinear regression was used to investigate the relationship between vegetation indices, yield results, and quality parameters from UAV NGB images.

## 5. Conclusions

Grain yield increased with increasing crop growth rate, but with LAI, grain yield increased only to a certain extent, after which increasing LAI did not significantly affect grain yield. Sushi had maximum LAI on different dates for the control and fertiliser treatments. This showed that Sushi had an excellent capacity for LAI. LAI values on 15 June 2020 showed minimum average values for all hybrids, and it had a maximum average values on 23 July 2020. LAI had maximum performance between the average values treatments in Sushi, Armagnac, Loupiac, and DKC4792 on 15 June 2020. This study also provides insights for examining variably applied N doses using crop sensors and UAV remote-sensing platforms.

## Figures and Tables

**Table 1:** Variance analysis of LAI on yield parameters.

Parameters	Source	df	F-Value	*p* Value
Moisture	Hybrid	6	16.49	0.000
	NPK	2	0.30	0.740
	Sampling Date	3	0.90	0.443
	LAI	225	1.59	0.005
Protein	Hybrid	6	16.63	0.000
	NPK	2	64.92	0.000
	Sampling Date	3	0.98	0.405
	LAI	225	0.96	0.593
Oil	Hybrid	6	9.83	0.000
	NPK	2	1.11	0.335
	Sampling Date	3	0.28	0.843
	LAI	225	0.81	0.896
Starch	Hybrid	6	29.64	0.000
	NPK	2	8.22	0.000
	Sampling Date	3	0.61	0.609
	LAI	225	0.97	0.585
Yield	Hybrid	6	1.89	0.089
	NPK	2	26.59	0.000
	Sampling Date	3	0.93	0.431
	LAI	225	1.01	0.493

**Table 2:** Factor analysis on yield parameters.

Variable	Factor 1	Factor 2	Factor 3	Factor 4	Factor 5	Communality
NPK	0.904	0.157	−0.056	−0.062	0.189	0.884
hybrid	0.159	−0.749	0.481	−0.077	−0.131	0.840
LAI	0.667	−0.090	0.057	0.563	−0.454	0.980
Moisture	0.053	0.812	0.032	−0.321	−0.435	0.955
Protein	0.927	−0.024	−0.070	−0.253	0.061	0.933
Oil	−0.154	−0.010	−0.898	0.271	0.031	0.904
Starch	−0.264	0.582	0.574	0.410	0.247	0.969
Yield	0.876	0.193	0.015	0.108	0.217	0.863
Variance	3.0096	1.6302	1.3792	0.7475	0.5614	7.3278
% Var	0.376	0.204	0.172	0.093	0.070	0.916

**Table 3:** Regression analysis on yield parameters.

	F-Value	*p*-Value	Regression Equation
Yield	185.72	0.000	Yield = 5868 + 0.01391 NPK − 0.1111 hybrid − 0.1332 sampling date + 1.943 LAI
Starch	5.90	0.000	Starch = 133 − 0.000880 NPK − 0.0498 hybrid − 0.00154 sampling date + 0.0224 LAI
Oil	13.56	0.000	Oil = 109.7 − 0.000332 NPK − 0.02859 hybrid − 0.00242 sampling date + 0.0353 LAI
Protein	208.46	0.000	Protein = 403 + 0.003314 NPK + 0.01192 hybrid − 0.00904 sampling date + 0.1319 LAI
Moisture	20.13	0.000	Moisture = −207 + 0.000893 NPK − 0.07302 hybrid + 0.00511 sampling date − 0.0745 LAI

**Table 4:** Regression analysis on vegetation index values and parameters.

Parameters	r	r^2^	Significance
BNDVI–yield	0.265744238	0.07062	**
BNDVI–oil	0.092422941	0.008542	NS
BNDVI–protein	0.3591657	0.129	***
BNDVI–starch	0.145567854	0.02119	NS
ENDVI–yield	0.723187389	0.523	***
ENDVI–oil	0.079906195	−0.006385	NS
ENDVI–protein	0.688694417	0.4743	***
ENDVI–starch	0.099599197	−0.00992	NS
GNDVI–yield	0.617413962	0.3812	***
GNDVI–oil	0.083486526	−0.00697	NS
GNDVI–protein	0.462709412	0.2141	***
GNDVI–starch	0.110136279	0.01213	NS
BNDVI*ENDVI~yield	0.629046898	0.3957	***
BNDVI*ENDVI~oil	0.044888751	−0.002015	NS
BNDVI*ENDVI~protein	0.629364759	0.3961	***
BNDVI*ENDVI~starch	0.059472683	−0.003537	NS
GNDVI*ENDVI~yield	0.259826865	0.06751	**
GNDVI*ENDVI~oil	0.106957936	0.01144	NS
GNDVI*ENDVI~protein	0.36	0.1296	***
GNDVI*ENDVI~starch	0.131529464	0.0173	NS
BNDVI*GNDVI*ENDVI~yield	0.272836948	0.07444	**
BNDVI*GNDVI*ENDVI~oil	0.099342841	0.009869	NS
BNDVI*GNDVI*ENDVI~protein	0.364142829	0.1326	***
BNDVI*GNDVI*ENDVI~starch	0.128257553	0.01645	NS

Significant codes: 0 ‘***’ 0.001 ‘**’ 0.01 ‘*’, NS: non-significant.

**Table 5:** LSD test on parameters.

Hybrid	Yield	Group	Hybrid	BNDVI	Group
SY Minerva	10.886453	a	Fornad	0.1387226	a
DKC4792	10.681877	ab	P0217	0.1375380	ab
Sushi	10.232131	abc	Loupiac	0.1368377	ab
Fornad	10.111388	abc	DKC4792	0.1367105	abc
Loupiac	9.825149	bc	Sushi	0.1355918	bc
P0217	9.393235	c	Armagnac	0.1355891	bc
Armagnac	9.343470	c	SY Minerva	0.1341214	c
Hybrid	Oil	Group	Hybrid	ENDVI	Group
Sushi	3.190833	a	Fornad	0.06731359	a
P0217	3.117500	a	Loupiac	0.06690510	a
Fornad	3.087500	ab	DKC4792	0.06676005	ab
SY Minerva	2.934167	bc	Sushi	0.06655601	ab
Loupiac	2.908333	c	Armagnac	0.06627764	ab
Armagnac	2.835000	c	P0217	0.06438019	b
DKC4792	2.834167	c	SY Minerva	0.06187965	c
Hybrid	Protein	Group	Hybrid	GNDVI	Group
SY Minerva	6.093333	a	P0217	0.1602840	a
Sushi	6.023333	ab	SY Minerva	0.1578918	ab
Armagnac	5.945833	b	Fornad	0.1564729	b
Loupiac	5.697500	c	DKC4792	0.1529463	c
DKC4792	5.665000	c	Loupiac	0.1529081	c
Fornad	5.530833	d	Armagnac	0.1514125	c
P0217	5.384167	e	Sushi	0.1507901	c
Hybrid	Starch	Group			
Fornad	66.06917	a			
Loupiac	65.99750	a			
P0217	65.93167	a			
SY Minerva	65.55750	b			
DKC4792	65.40917	bc			
Armagnac	65.20333	c			
Sushi	64.55750	d			

Treatments with the same letter are not significantly different.

**Table 6:** LSD test on nitrogen levels.

Parameters	Nitrogen Level		Group
Yield	0	5.357643	b
	60	11.871939	a
	150	12.973433	a
Protein	0	5.165000	c
	60	5.748571	b
	150	6.375000	a
Starch	0	65.52500	ab
	60	65.73821	a
	150	65.33357	b
ENDVI	0	0.07021143	a
	60	0.06441199	b
	150	0.06255039	b
GNDVI	0	0.1463726	b
	60	0.1567136	a
	150	0.1609305	a

Treatments with the same letter are not significantly different.

**Table 7:** Soil parameters in this experiment.

Fertilisation Levels	pH (KCl 1:2,5)	K_A_	Salt Content [m/m%]	CaCO_3_ [m/m%]	Organic Matter [m/m%]	Nitrogen [mg/kg]	Magnesium [mg/kg]	Potassium Oxide [mg/kg]	Phosphorus Pentoxide [mg/kg]
0	6.15	38.56	<0.02	<0.1	2.16	1.17	362.30	185.28	52.90
1	5.70	40.28	<0.02	<0.1	2.23	2.30	346.15	277.44	146.65
2	5.57	36.81	<0.02	<0.1	2.02	2.11	359.00	277.02	129.12

Notes: pH (KCL), potassium chloride soluble pH; K_A_, Arany’s plasticity index. Fertilisation levels: 0: control, 1: 120 kg ha^−1^, 2: 300 kg ha^−1^.

## Data Availability

All data supporting the conclusions of this article are included in this article.
